# Supplementary material for: IL-27 maintains cytotoxic Ly6C+ γδ T cells that arise from immature precursors
Source: EMBO J. 2024 May 30;43(14):5. doi: 10.1038/s44318-024-00133-1 (PMC11251046; doi:10.1038/s44318-024-00133-1)
Supplement: Supplementary file 4 — Figure 7I Source Data [file 44318_2024_133_MOESM4_ESM.pdf]

Figure 7I

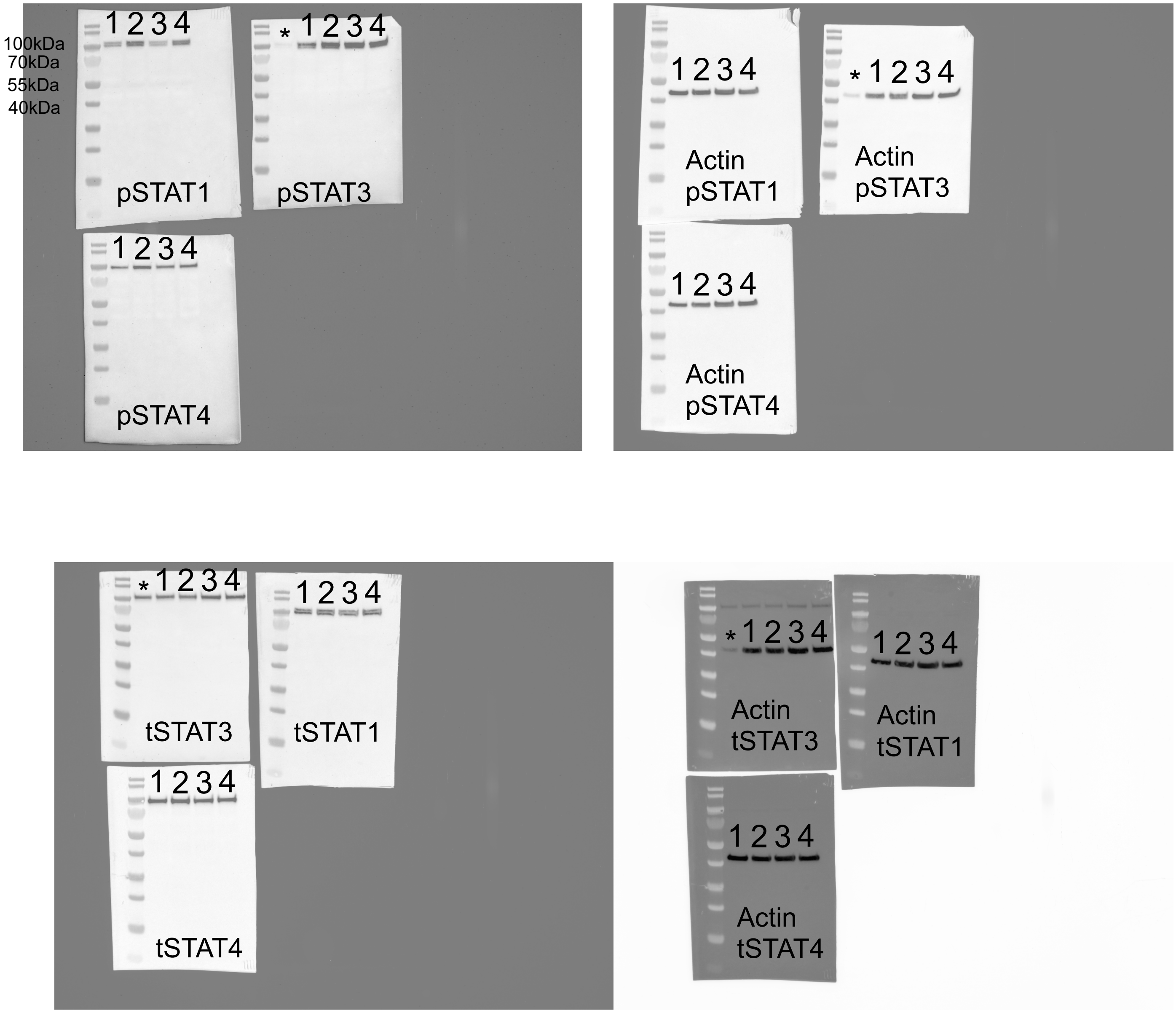

1: Ly6C^{-} $\gamma\delta$T cells IL-2 and IL-15
2: Ly6C^{-} $\gamma\delta$T cells IL-2, IL-15 and IL-27
3: Ly6C^{+} $\gamma\delta$T cells IL-2 and IL-15
4: Ly6C^{+} $\gamma\delta$T cells IL-2, IL-15 and IL-27
*: for pSTAT3/tSTAT3 A549 cell lysate
