# Supplementary material for: IL-27 maintains cytotoxic Ly6C+ γδ T cells that arise from immature precursors
Source: EMBO J. 2024 May 30;43(14):5. doi: 10.1038/s44318-024-00133-1 (PMC11251046; doi:10.1038/s44318-024-00133-1)
Supplement: Supplementary file 6 — Expanded View Figures [file 44318_2024_133_MOESM6_ESM.pdf]

Expanded View Figures

Figure EV1. Expression of cytotoxic markers and TCR usage by CD27$^+$Ly6C$^-$ and CD27$^+$Ly6C$^+$ γδ T cells.

(**A**) Flow cytometry plots for expression of indicated proteins in CD27$^+$Ly6C$^-$ and CD27$^+$Ly6C$^+$ γδ T cells from spleen of naive FVB/n mice. Fluorescence minus one (FMO) controls were used to set gating. (**B**) Flow cytometry plots of TCR chain usage on CD27$^+$ γδ T cells and Ly6C expression of each population.

 © The Author(s)

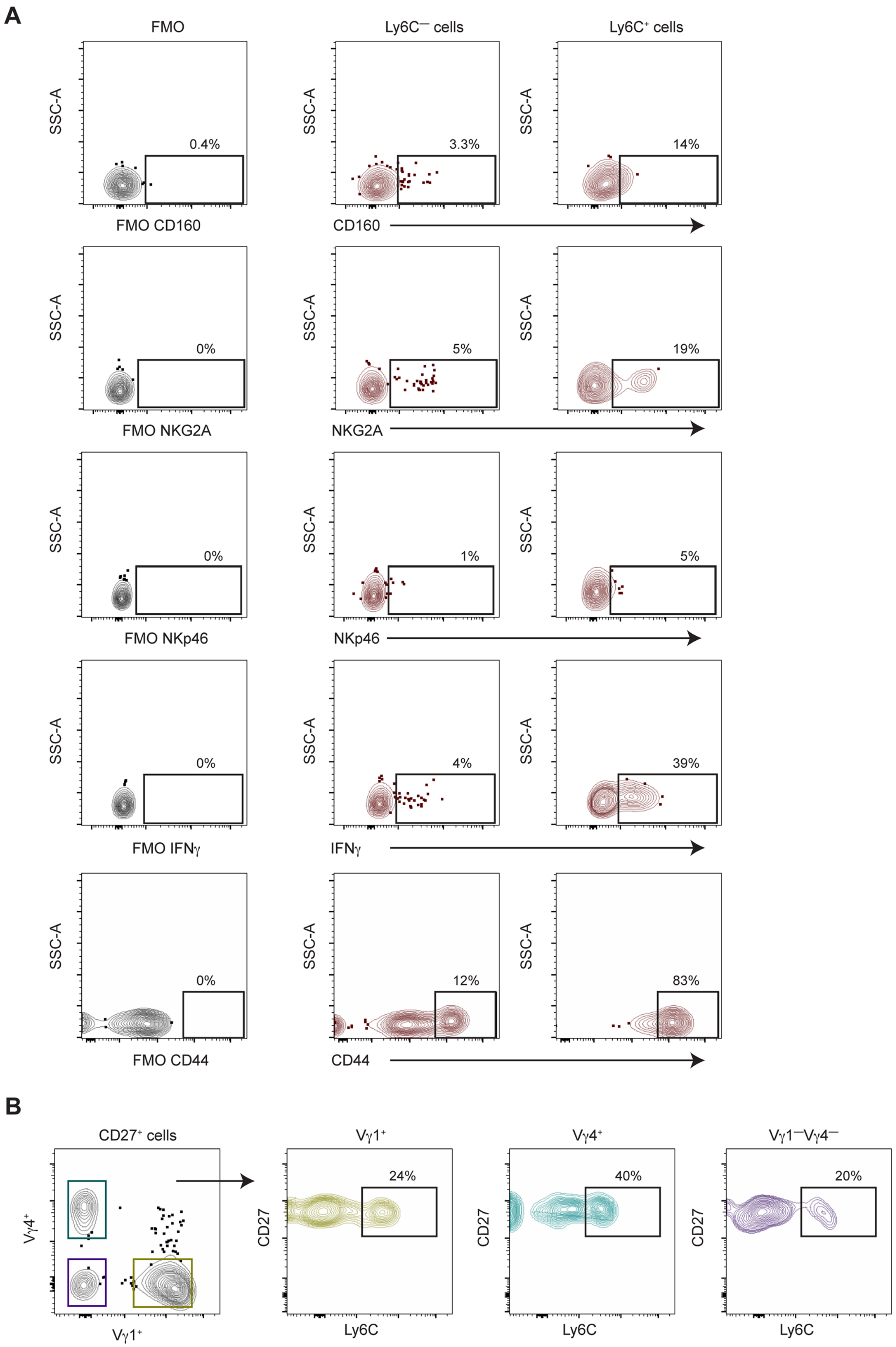
A
FMO
Ly6C⁻ cells
Ly6C⁺ cells
SSC-A
0.4%
3.3%
14%
FMO CD160
CD160
0%
5%
19%
FMO NKG2A
NKG2A
0%
1%
5%
FMO NKp46
NKp46
0%
4%
39%
FMO IFNγ
IFNγ
0%
12%
83%
FMO CD44
CD44
B
CD27⁺ cells
Vγ1⁺
Vγ4⁺
Vγ1⁻Vγ4⁻
24%
40%
20%
CD27
Ly6C

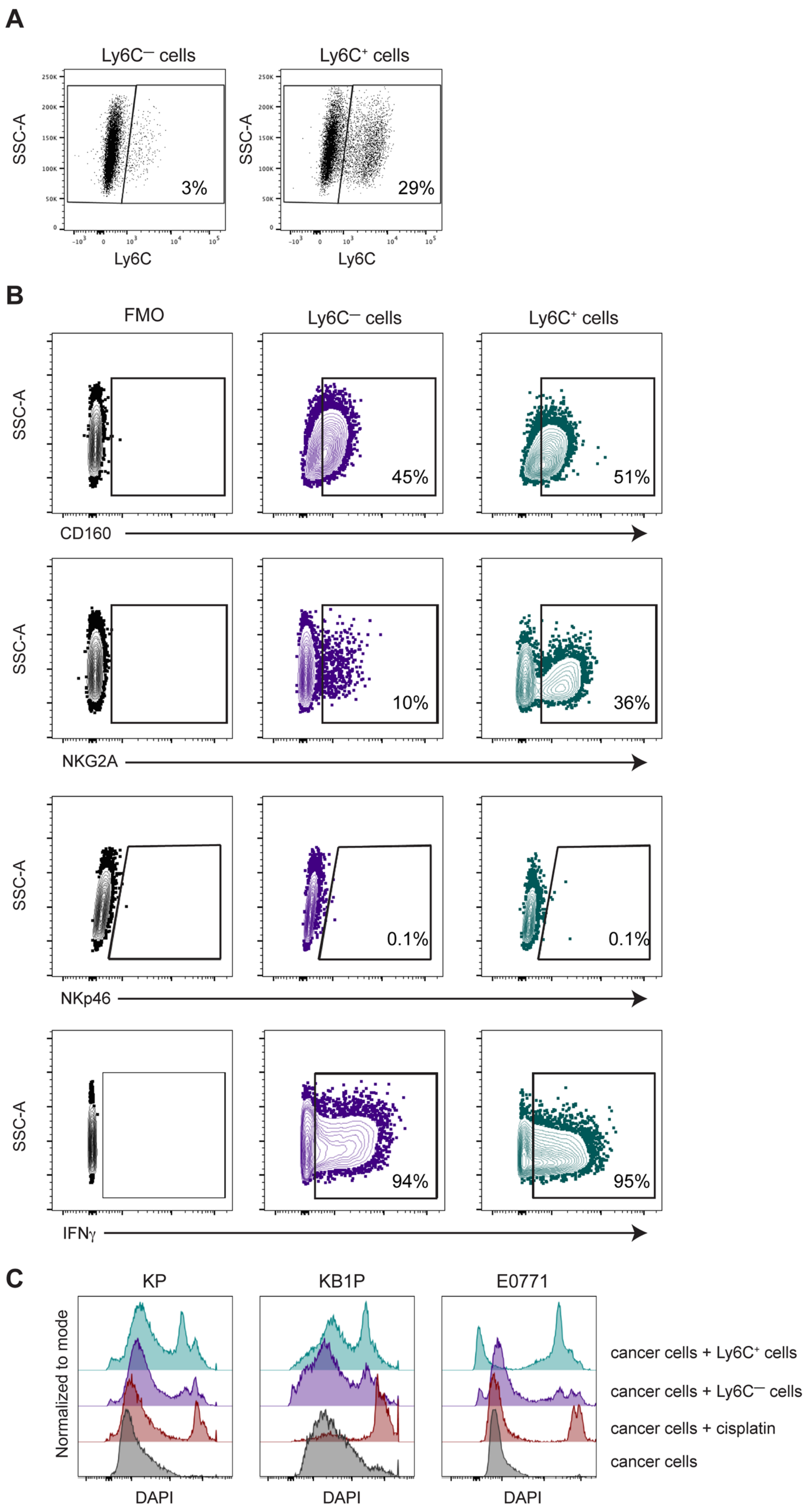
A
Ly6C⁻ cells
Ly6C⁺ cells
SSC-A
3%
29%
Ly6C
B
FMO
Ly6C⁻ cells
Ly6C⁺ cells
SSC-A
45%
51%
CD160
10%
36%
NKG2A
0.1%
0.1%
NKp46
94%
95%
IFNγ
C
KP
KB1P
E0771
Normalized to mode
DAPI
cancer cells + Ly6C⁺ cells
cancer cells + Ly6C⁻ cells
cancer cells + cisplatin
cancer cells

 © The Author(s)

Figure EV2. Phenotyping and cancer cell killing ability of CD27^{+}Ly6C^{-} and CD27^{+}Ly6C^{+} γδ T cells.

(**A**) Flow cytometry plots of Ly6C expression on sorted CD27^{+}Ly6C^{-} and CD27^{+}Ly6C^{+} γδ T-cell subsets expanded ex vivo over 4 days in the presence of CD3/CD28 Dynabeads, IL-2, and IL-15. (**B**) Flow cytometry plots for expression of indicated proteins in expanded CD27^{+}Ly6C^{-} and CD27^{+}Ly6C^{+} γδ T cells. Fluorescence minus one (FMO) controls were used to set gating. (**C**) Representative histograms of cancer cell death measured by DAPI uptake after co-culture with ex vivo-expanded CD27^{+}Ly6C^{-} and CD27^{+}Ly6C^{+} γδ T cells or cisplatin treatment.

© The Author(s)

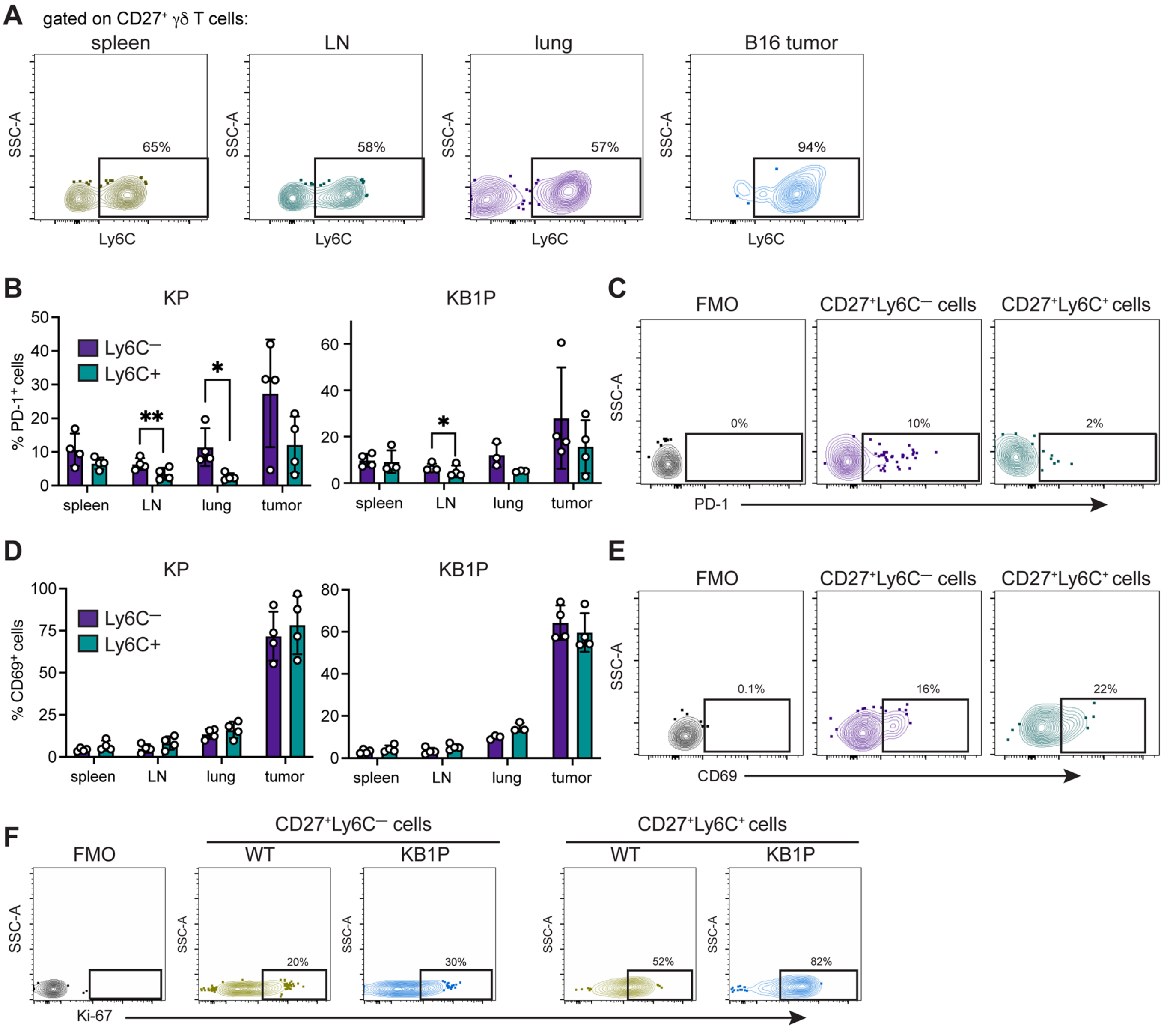

Figure EV3. Ly6C and Ki-67 expression in tumor-associated CD27$^+$Ly6C$^-$ and CD27$^+$Ly6C$^+$ γδ T cells.

(**A**) Flow cytometry plots of Ly6C expression on CD27$^+$ γδ T cells in indicated tissue from B16-F1 tumor-bearing mice. (**B**) Frequency of PD-1$^+$ cells in CD27$^+$Ly6C$^-$ and CD27$^+$Ly6C$^+$ γδ T cells in indicated tissues of tumor-bearing KP and KB1P mice ($n = 3$–4). Each dot represents one tumor-bearing mouse. *$P < 0.05$, **$P < 0.01$ (paired t test). Data are represented as mean ± SD. (**C**) Representative flow cytometry plots of PD-1 expression on CD27$^+$Ly6C$^-$ and CD27$^+$Ly6C$^+$ γδ T cells from lungs of tumor-bearing KP mice. (**D**) Frequency of CD69$^+$ cells in CD27$^+$Ly6C$^-$ and CD27$^+$Ly6C$^+$ γδ T cells in indicated tissues of tumor-bearing KP and KB1P mice ($n = 3$–4). Each dot represents one tumor-bearing mouse. Data are represented as mean ± SD. (**E**) Representative flow cytometry plots of CD69 expression on CD27$^+$Ly6C$^-$ and CD27$^+$Ly6C$^+$ γδ T cells from lungs of tumor-bearing KP mice. (**F**) Flow cytometry plots of Ki-67 expression on CD27$^+$Ly6C$^-$ and CD27$^+$Ly6C$^+$ γδ T cells from FVB/n WT and KB1P tumor-bearing mice.

© The Author(s)

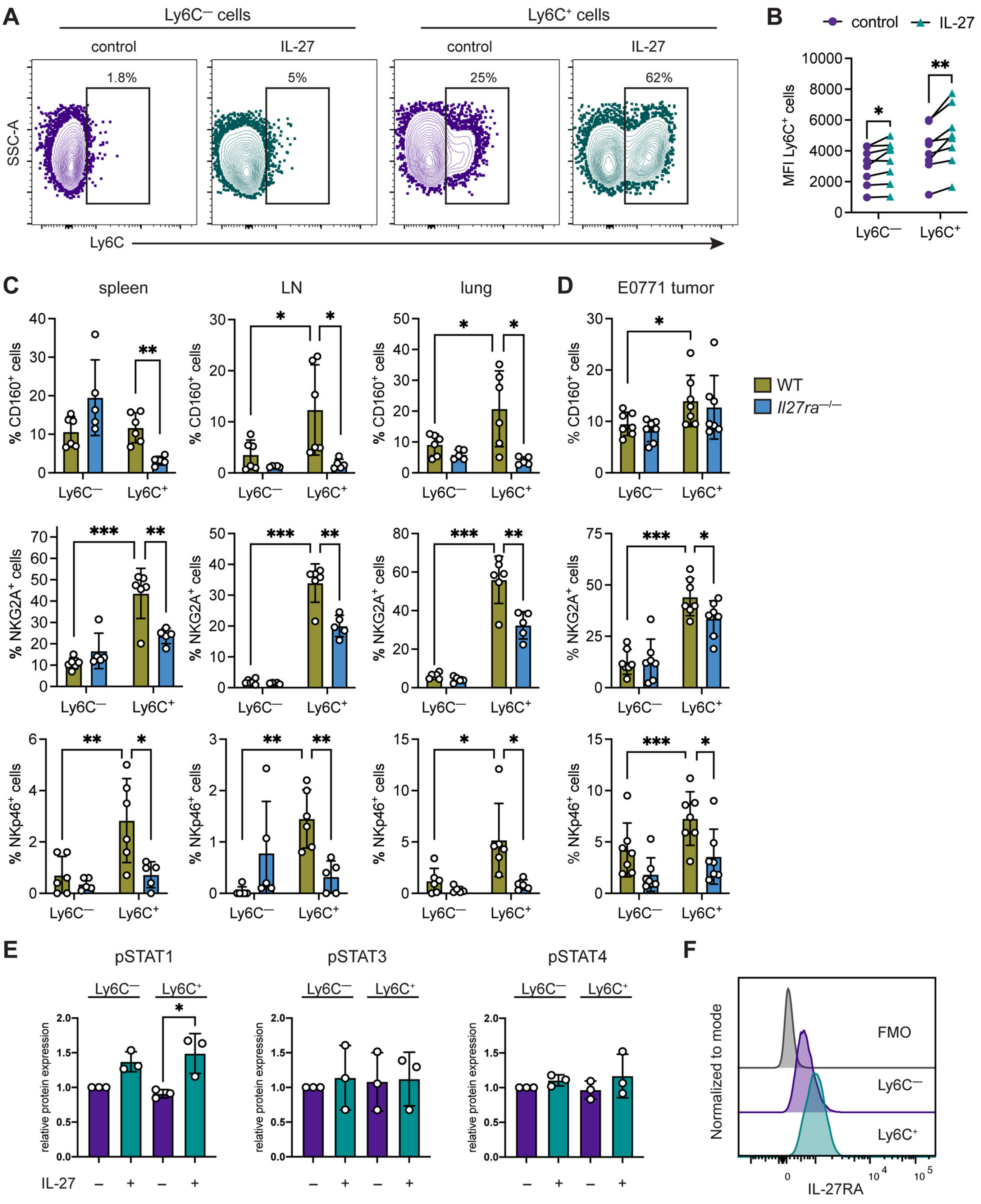
A
Ly6C⁻ cells
Ly6C⁺ cells
control
IL-27
control
IL-27
1.8%
5%
25%
62%
SSC-A
Ly6C
B
control
IL-27
MFI Ly6C⁺ cells
10000
8000
6000
4000
2000
0
Ly6C⁻
Ly6C⁺
C
spleen
LN
lung
D
E0771 tumor
% CD160⁺ cells
% NKG2A⁺ cells
% NKp46⁺ cells
WT
Il27ra⁻/⁻
E
pSTAT1
pSTAT3
pSTAT4
relative protein expression
IL-27
F
FMO
Ly6C⁻
Ly6C⁺
Normalized to mode
IL-27RA

Figure EV4. IL-27 regulates CD27$^+$Ly6C$^+$ γδ T cells.

(**A**) Flow cytometry plots of Ly6C expression on sorted CD27$^+$Ly6C$^-$ and CD27$^+$Ly6C$^+$ γδ T-cell subsets expanded ex vivo over 4 days in the presence of CD3/CD28 Dynabeads, IL-2, and IL-15, as well as IL-27 where indicated. (**B**) Median fluorescence intensity (MFI) of Ly6C expression after gating on Ly6C$^+$ cells within sorted CD27$^+$Ly6C$^-$ or CD27$^+$Ly6C$^+$ γδ T-cell subsets expanded ex vivo over 4 days with CD3/CD28 beads, IL-2, and IL-15 (control), with IL-27 as indicated. Individual replicates are shown as pairs ($n = 8$). Each dot represents expanded cells from pooled LNs and spleens of 6 mice. *$P < 0.05$, **$P < 0.01$ (paired *t* test). (**C**, **D**) Proportion of CD160, NKG2A, and NKp46-expressing Ly6C$^-$ and Ly6C$^+$ cells in indicated tissues from C57BL/6 WT ($n = 6$ tumor-free, 7 tumor-bearing) or *Il27ra*$^{-/-}$ ($n = 5$ tumor-free, 7 tumor-bearing) mice. Each dot represents one mouse *$P < 0.05$, **$P < 0.01$, ***$P < 0.001$ (unpaired *t* test). Each dot represents one mouse. Data are represented as mean ± SD. *$P < 0.05$ (unpaired or paired student *t* test). (**E**) Densitometry graphs representing relative protein expression of indicated phosphorylated (p) STAT proteins after in vitro culture of CD27$^+$Ly6C$^-$ and CD27$^+$Ly6C$^+$ γδ T cells in the presence or absence of IL-27 from cells from (**A**). First condition was set to 1 in order to normalize between independent biological replicates ($n = 3$). Each dot represents one independent in vitro culture from a pool of 6 mice. Data are represented as mean ± SD. *$P < 0.05$ (repeated measures one-way ANOVA followed by Tukey's posthoc test). (**F**) Representative histograms of IL-27RA expression in CD27$^+$Ly6C$^-$ and CD27$^+$Ly6C$^+$ γδ T cells from lymph node tissue of C57BL/6 WT mice.

© The Author(s)

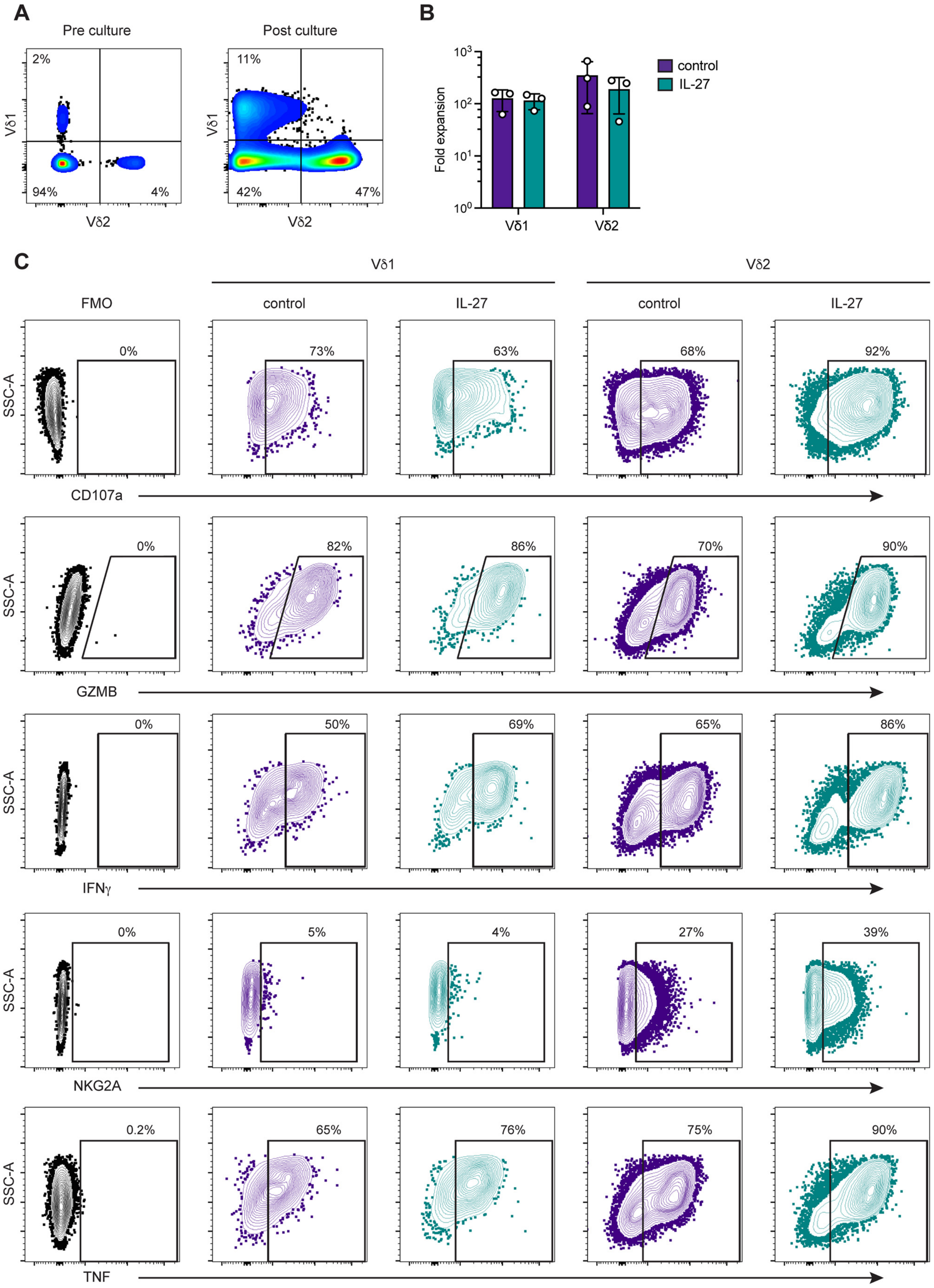
A
Pre culture
Post culture
2%
94%
4%
11%
42%
47%
Vδ1
Vδ2
B
Fold expansion
control
IL-27
Vδ1
Vδ2
C
Vδ1
Vδ2
FMO
control
IL-27
control
IL-27
SSC-A
CD107a
0%
73%
63%
68%
92%
GZMB
0%
82%
86%
70%
90%
IFNγ
0%
50%
69%
65%
86%
NKG2A
0%
5%
4%
27%
39%
TNF
0.2%
65%
76%
75%
90%

Figure EV5. Human Vδ2 cells respond to IL-27 stimulation.

(**A**) Flow cytometry plots of Vδ1 and Vδ2 T cells before expansion (left) and after culture for 14 days with IL-2 and IL-15. (**B**) Fold expansion of human Vδ1 and Vδ2 cells with IL-2 and IL-15 (control) or IL-2, IL-15, and IL-27 ($n = 3$ human PBMC donors/group). Data are represented as mean ± SD. (**C**) Flow cytometry plots of ex vivo-expanded cells from (**A**). Live $CD3^{+}$ cells were gated on Vδ1 and Vδ2. Expression of CD107a, Granzyme B (GZMB), IFNγ, NKG2A and TNF was measured on Vδ1 and Vδ2 cells for both culture conditions. FMO controls were used to set gating.

 © The Author(s)
